# Draft Genome Sequence of Kytococcus schroeteri Strain H01, Isolated from Human Skin

**DOI:** 10.1128/MRA.01081-19

**Published:** 2019-10-03

**Authors:** Amine M. Boukerb, Magalie Robert, Nataliya A. Teteneva, Natalia D. Danilova, Marina V. Zhurina, Sergey V. Mart’yanov, Vladimir K. Plakunov, Marc G. J. Feuilloley, Andrei V. Gannesen

**Affiliations:** a Laboratory of Microbiology Signals and Microenvironment LMSM EA4312, University of Rouen, Evreux, France; b Department of Systems and Synthetic Microbiology, Max Planck Institute for Terrestrial Microbiology & LOEWE Center for Synthetic Microbiology (SYNMIKRO), Marburg, Germany; c Laboratory of Viability of Microorganisms, FRS Fundamentals of Biotechnology of the Russian Academy of Sciences, Moscow, Russia; University of Rochester School of Medicine and Dentistry

## Abstract

Kytococcus schroeteri strain H01 was isolated from the skin of a healthy volunteer who underwent erythromycin treatment for a skin disorder 1 year prior. The draft genome consists of 2.38 Mb, a G+C content of 73.06%, and 2,221 protein coding sequences. This is the first genome characterization of a K. schroeteri strain isolated from human skin.

## ANNOUNCEMENT

Kytococcus schroeteri inhabits human skin, and the genus name literally translates as “cutaneous coccus” (1). It was first isolated in 2002 from the blood of a patient with endocarditis (2). *K. schroeteri* is an aerobic, Gram-positive, nonmotile, and nonencapsulated coccus (diameter, 1.1 to 1.5 μm). Cells mostly occur singly but can also be organized in pairs, tetrads, and even occasionally in clusters (2). Here, we report the draft genome sequence of *K. schroeteri* H01, which was isolated from the skin of a healthy volunteer who had undergone antibiotic treatment for misdiagnosed atypical skin herpes simplex disorder 1 year previously. The isolated strain was characterized as *K. schroeteri* by 16S rRNA gene sequencing (3).

*K. schroeteri* H01 genomic DNA was extracted from an overnight culture in 25 ml LB broth with the GeneJet genomic DNA purification kit per the manufacturer’s protocol with no modifications (Thermo Scientific, France). Genomic DNA was diluted to a concentration of 0.2 ng/μl as quantified by a double-stranded DNA (dsDNA) high-sensitivity kit on a Qubit fluorometer (Thermo Fisher Scientific, USA). Library preparation of 1 ng DNA was performed using the Nextera XT DNA library preparation kit per the manufacturer’s protocol (Illumina, San Diego, CA, USA). The library was sequenced on the Illumina MiSeq platform (LMSM Genomics, Rouen Normandy University, France) using the MiSeq version 3 reagent kit (2 × 250 bp), and 2,524,220 paired-end reads were generated.

Raw paired-end reads were quality filtered using Trim Galore v.0.6.2 (4) with set default parameters. FastQC v.0.11.8 (5) with default parameters was used to check the quality of the 2,161,744 validated reads. Assembly of paired-end reads was done *de novo* using Unicycler v.0.4.7 with default settings (6). Quast v.5.0.0 was used to check assembly consistency, such as number of contigs, G+C content, *N*_50_ value, and the total size (7). Annotations were done using the Prokka pipeline v1.13.4 with default parameters (8).

The draft genome contains 179 contigs with an *N*_50_ value of 23,118 bp and a largest contig size of 105,946 bp. The total estimated size was 2,381,857 bp, with an average G+C content of 73.06%. The draft genome had 2,221 predicted protein coding sequences, 48 tRNA genes, and 3 rRNA genes. The 16S rRNA gene showed 100% identity with that of *K. schroeteri* strain Muenster 2000^T^.

### Data availability.

The draft genome sequence of *K*. *schroeteri* H01 obtained in this whole-genome shotgun project has been deposited at DDBJ/ENA/GenBank under the accession number VHHR00000000. The version described in this paper is the first version, VHHR01000000. The raw sequencing data have been deposited in the same database under the accession number SRR9323812. The BioSample and BioProject numbers are SAMN12090546 and PRJNA549517, respectively.
